# Montreal Veterinary Medical Association

**Published:** 1896-05

**Authors:** 


### MONTREAL VETERINARY MEDICAL ASSOCIATION.

*Resolutions on the death of Dr. Campbell, Class '92 (McGill).* *Whereas*, it has pleased an allwise Providence to remove from our midst a professional brother in the person of the late Dr. Donald Campbell; be it *Resolved*, That this Association by his death loses an honored member, esteemed alike for his devotion to the cause of the profession, his upright conduct and genial nature; and be it *Resolved*, That a copy of these resolutions be spread upon the records of the Association; and be it *Resolved*, That a copy of these resolutions be forwarded to the family of our deceased brother. Signed on behalf of the Association, M. A. Dawes, D.V.S.; F. W. Kee, J. Anderson Ness, *Committee*.

